# Forward With Dementia: Co‐designing resources adapted for the Chinese, South Asian and Italian communities

**DOI:** 10.1002/alz70858_101681

**Published:** 2025-12-25

**Authors:** Carrie A McAiney, Emma Bender, Dana Kirkbride, Dana Zummach, Christine Pellegrino

**Affiliations:** ^1^ University of Waterloo, Waterloo, ON, Canada; ^2^ Schlegel‐UW Research Institute for Aging, Waterloo, ON, Canada

## Abstract

**Background:**

Stigma is a common experience for people living with dementia, is associated with many negative consequences including discrimination and social exclusion, and can serve as a barrier to accessing services for people living with dementia. Unfortunately, dementia‐related stigma may be even more likely to occur in ethno‐racial communities. Dementia resources that are culturally relevant to ethno‐racial communities and written in the languages read in these communities are lacking. Forward With Dementia (FWD) is an initiative that aims to combat stigma and raise awareness about living well with dementia. To begin to address the gap in culturally relevant information and resources, the Canadian FWD team co‐designed resources that were adapted for the Chinese, South Asian and Italian communities.

**Method:**

Co‐design teams were established for each ethno‐racial community. Teams included family/friend care partners and health and social care providers. Unfortunately, we were unable to recruit people living with dementia to participate, likely due to increased dementia‐related stigma and the tendency of later diagnosis in these communities. Content from the existing FWD site served as the basis for the resources. We met monthly with each co‐design team to adapt and review the resources. Pictures, symbols, personal stories, and examples relevant to each community were used in the design of the resources. Once finalized, the resources were translated into relevant languages.

**Result:**

Over a 1‐year period, a total of 187 newly adapted resources were created. This included 13 core resources and 1‐2 stories by care partners for each community. Chinese resources were translated into Simplified and Traditional Chinese, South Asian resources were translated into Punjabi, Hindi and Urdu, and Italian resources were translated into Italian. The resources are also available in English and French. During a 3‐month marketing campaign to promote the resources, over 157,000 individuals were reached with 4300+ views of the resources and 1400+ downloads.

**Conclusion:**

The new culturally‐adapted and translated FWD resources address a significant gap in support for people with dementia and care partners from ethno‐racial communities.

